# Light-induced carotenogenesis enhances growth and high hydrostatic pressure tolerance in a deep-sea bacterium by reducing intracellular ROS

**DOI:** 10.1128/aem.00658-26

**Published:** 2026-08-28

**Authors:** Jin Lin, Xue-Gong Li, Wei-Jia Zhang, Hai-Rong Fang, Guan-Yuan Zhang, Long-Fei Wu

**Affiliations:** 1 Laboratory of Deep-Sea Microbial Cell Biology, Institute of Deep-sea Science and Engineering, Chinese Academy of Sciences 383875, Sanya, People's Republic of China; 2 University of Chinese Academy of Sciences 74519, Beijing, People's Republic of China; 3 International Associated Laboratory of Evolution and Development of Magnetotactic Multicellular Organisms, CNRS-Marseille/CAS-Sanya, Sanya, China; 4 Institution of Deep-sea Life Sciences, IDSSE-BGI, Hainan Deep-sea Technology Laboratory 383875, Sanya, Hainan, People's Republic of China; 5 CNRS, LCB, IMM, IM2B, Aix Marseille Univ, Marseille, France; University of Delaware, Lewes, Delaware, USA

**Keywords:** deep-sea hydrothermal vent, light, carotenoids, reactive oxygen species, high hydrostatic pressure

## Abstract

Deep-sea hydrothermal vents emit geothermal light, yet its ecological role in shaping microbial adaptation remains largely unexplored. To investigate this, we designed a custom cultivation system that simultaneously applies high hydrostatic pressure (HHP) and light and used it to isolate 12 bacterial strains from active chimney fragments collected at the Kairei hydrothermal field. All 12 isolates exhibited enhanced growth under light exposure. Among them, strain SY138, identified as a novel *Microbacterium* species, was selected for mechanistic investigation. Our results demonstrate that light exposure directly induces carotenoid biosynthesis in SY138, which in turn reduces intracellular reactive oxygen species (ROS) levels. Inhibition of carotenoid synthesis using fosmidomycin abolished both the ROS-lowering effect and the light-dependent growth promotion, confirming that carotenoids are essential for this physiological response. Notably, while HHP severely inhibited growth by elevating intracellular ROS, light-induced carotenogenesis effectively counteracted this oxidative stress and alleviated HHP-induced growth inhibition. Collectively, these findings uncover a previously unrecognized adaptive strategy, wherein deep-sea hydrothermal vent bacteria exploit geothermal light as an environmental signal to activate carotenoid-based antioxidant defenses, thereby enhancing their survival under HHP conditions. This study highlights a previously underappreciated role of light in microbial stress tolerance in extreme deep-sea ecosystems.

**IMPORTANCE:**

Deep-sea hydrothermal vents harbor thriving ecosystems under extreme pressure and perpetual darkness, yet they have long been considered to be driven solely by chemosynthesis. The discovery of geothermal light has raised fundamental questions regarding its ecological role. Using a specialized cultivation system that simulates *in situ* high pressure and illumination, we isolated multiple light-responsive bacterial strains from a deep-sea vent, substantially expanding the known diversity of such microorganisms. Using *Microbacterium* sp. SY138 as a model, we uncovered a previously unrecognized adaptive strategy: these bacteria exploit light not as an energy source but as an environmental cue to preemptively activate carotenoid-based antioxidant defenses. This light-induced mechanism counteracts oxidative stress imposed by high pressure, a ubiquitous deep-sea stressor. Our findings reveal an unexpected interplay between light and pressure in the deep-sea environment, suggesting that light sensing could represent a more widespread and ecologically significant adaptive trait than previously recognized.

## INTRODUCTION

The deep sea, characterized by high hydrostatic pressure (HHP), low temperature, and aphotic conditions, represents one of the largest and most extreme habitats on Earth. Among these factors, HHP is a pervasive physicochemical constraint that dictates the distribution, physiology, and metabolic activity of microorganisms (1). To cope with the deleterious effects of HHP, which include protein denaturation, membrane rigidification, and DNA damage, deep-sea bacteria have evolved sophisticated adaptation strategies, such as the incorporation of unsaturated fatty acids into membranes and the accumulation of compatible solutes (2–4). In addition, they also modulate gene expression programs and activate stress-responsive loci in response to pressure changes (5, 6). Despite these well-recognized adaptations, emerging evidence indicates that exposure to HHP often triggers an imbalance in cellular redox homeostasis, leading to the overproduction of reactive oxygen species (ROS) (7, 8). This oxidative stress is now recognized as a critical, yet often underappreciated, component of cellular damage under high-pressure conditions (9, 10).

Although the deep sea is traditionally viewed as a realm devoid of sunlight, recent explorations have revealed that hydrothermal vents can harbor sources of geologically generated thermal radiation (11–13). Intriguingly, deep-sea bacteria, particularly those isolated from hydrothermal vents, have been found to possess photosynthetic pigments (14, 15), photosensory proteins (16, 17), and enzymes for carotenoid biosynthesis (18, 19). Carotenoids are best known for their photoprotective and antioxidant functions in photosynthetic and surface-dwelling organisms, where they quench singlet oxygen and scavenge free radicals (20). This distribution raises a compelling paradox: why do bacteria thriving in the permanent darkness of the deep sea retain functional light-sensing and carotenoid-producing machinery? We hypothesize that in these organisms, light may not serve as an energy source but rather as an environmental cue that triggers a protective antioxidant response against oxidative stress induced by HHP.

Carotenogenesis is known to be regulated by light in various bacterial lineages, such as *Myxococcus* and *Cyanobacteria* (21, 22). However, the physiological significance of light-induced carotenoid production in the context of deep-sea adaptation remains entirely unexplored. Specifically, it is unknown whether light exposure can stimulate carotenoid synthesis in deep-sea isolates, and if so, whether this light-primed accumulation of carotenoids can confer cross-protection against HHP stress by mitigating intracellular ROS accumulation. Addressing these questions is critical for understanding the integrated stress responses that enable microbial life in extreme environments.

To test this hypothesis, we employed a custom-designed HHP and light-coupled cultivation system to simulate deep-sea hydrothermal environments. From active chimney fragments collected at the Kairei hydrothermal field in the Central Indian Ridge, we isolated 12 microbial strains exhibiting light-enhanced growth under HHP conditions. Focusing on a novel species, *Microbacterium* sp. SY138, we investigated the mechanism underlying light-promoted growth. Our results indicate that light-stimulated growth of strain SY138 depends on carotenoid synthesis, which reduces intracellular ROS levels. Notably, while HHP inhibits growth by elevating ROS levels, light-induced carotenoid synthesis effectively lowers ROS and alleviates HHP-induced growth inhibition. These findings expand our understanding of how deep-sea microorganisms adapt to high-pressure environments.

## MATERIALS AND METHODS

### Sample collection

During the TS10-3 cruise of the research vessel “*Tan Suo Yi Hao*” in February 2019, active chimney fragments were collected at a depth of 2,495 m from the Kairei hydrothermal field (25.32 °S, 70.04 °E) in the Central Indian Ridge using the manned submersible “*Shen Hai Yong Shi*.” The chimney fragments were retrieved by the robotic arm of the submersible and placed in a sealed biobox filled with sterile seawater. Once onboard, the samples were crushed under sterile conditions and then stored anaerobically at 4°C until further processing.

### Enrichment and isolation conditions

**Fig 1:**
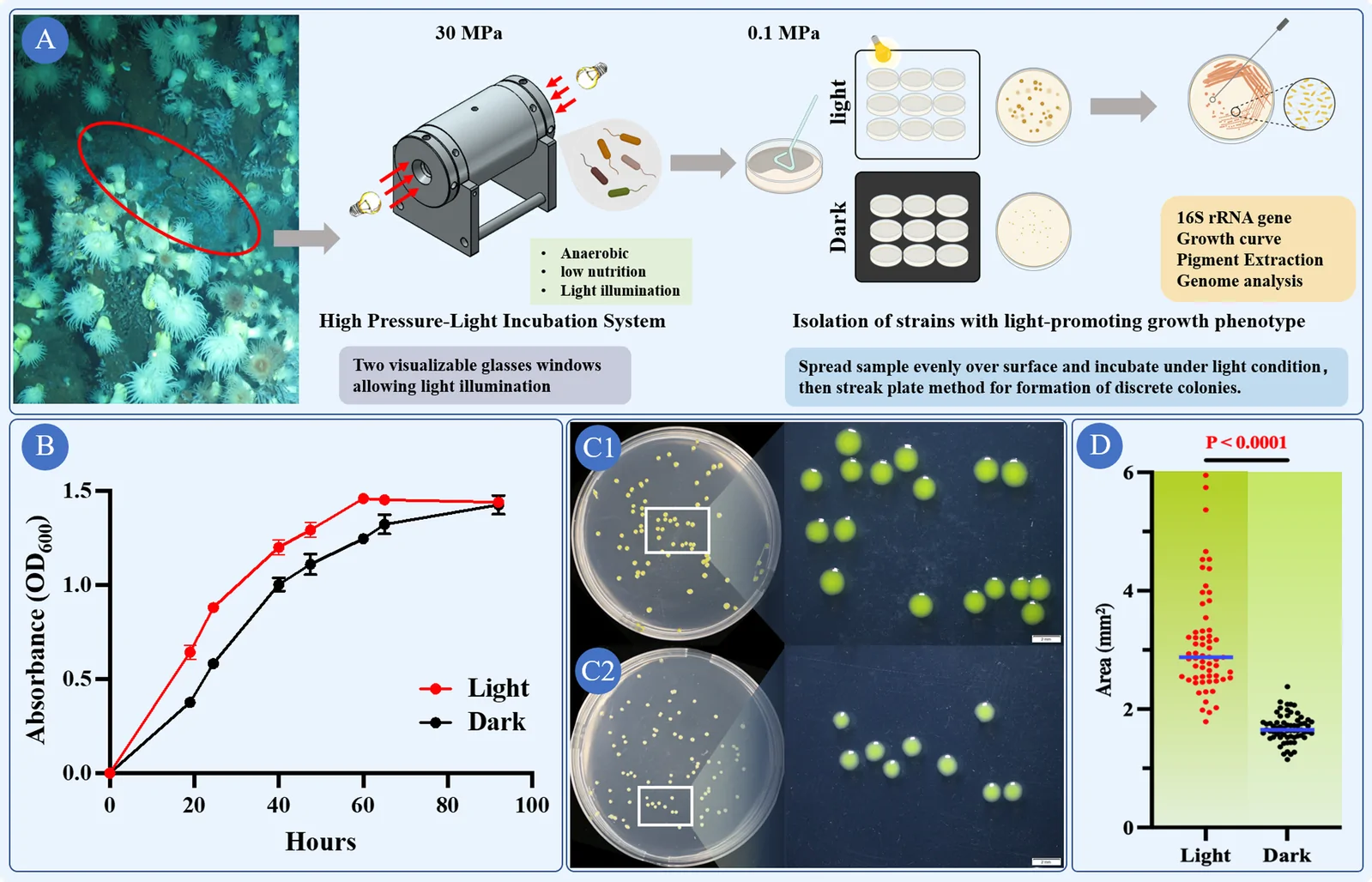
Enrichment and isolation of light-promoted strains. (**A**) Flowchart for the enrichment, isolation, and identification of light-promoted growth microorganisms. (**B**) Growth curve of *Microbacterium* sp. SY138 under light and dark conditions at 0.1 MPa. Data are presented as mean ± SD (*n* = 3). (**C**) Colony morphology of SY138 strain under light (**C1**) and dark (**C2**) conditions. (**D**) Colony size of SY138 strain under light and dark conditions. For statistical analysis, an unpaired *t*-test was used.

Enrichment under illuminated conditions was performed using high-pressure illumination (HPI) incubators developed by our laboratory, which feature two glass windows on opposite sides to allow light transmission. For the light treatment, HPI incubators were irradiated by halogen lamps positioned on both sides of the incubator (Fig. 1A). The light intensity was set to 1 mW/cm^2^ using a Powermeter instrument (PM100D, THORLABS, German). For the dark control, the glass windows of the HPI incubator were covered with black opaque tape. The stored chimney debris (about 2.0 g) was transferred into 160 mL anaerobic Medium 2 as previously described (23). Trace element solution, vitamin solution, and NaHCO_3_ were added after subsequently sterilized by autoclaving at 121°C for 20 min with N_2_ as the headspace gas. The medium was then distributed into two HPI incubators for light and dark incubation at 25°C under 30 MPa.

After approximately 1 month of incubation, the medium turned turbid under the light condition, suggesting that microbial growth was promoted under illumination. To obtain pure cultures, equal volumes of the light-treated culture were spread anaerobically on plates of Medium 2 solidified with agar (2%, wt/vol). The plates were then randomly divided into two groups: one group was placed under light conditions, while the other was wrapped in aluminum foil to exclude light and placed under the same ambient conditions. After growth at 25°C for 1 month at 0.1 MPa, single colonies with distinct morphologies and colors were selected from the light-exposed culture plates for further purification by repeated streaking (Fig. 1A). The purity of the isolates was subsequently confirmed through microscopic examination and 16S rRNA gene sequencing.

### Genome sequencing and assembly

For genomic DNA extraction, strain SY138 was cultivated in 2216E medium at 25°C. Cells were harvested during the late exponential phase by centrifugation at 4,500 × *g* for 20 min. Genomic DNA was extracted using the MagAttract DNA Kit (Qiagen) following the manufacturer’s protocol. Whole-genome sequencing was performed by BGI-Qingdao (Qingdao, China) using the BGI SEQ-500 platform and Nanopore sequencing systems. Clean reads were assembled with SOAPdenovo, and the G+C content was calculated using GC-Depth.

### Quantification of colony size

Strain *Microbacterium* sp. SY138 was inoculated into 2216E medium for activation. The resulting culture was diluted to an OD600 of approximately 0.05, thoroughly mixed, and evenly spread onto the surface of agar plates. The plates were incubated separately under light and dark conditions. For the light treatment, plates were incubated under a halogen lamp; for the dark control, plates were wrapped in aluminum foil and placed under the same lamp. After approximately 65 h of incubation, single colonies became visible on the plates. The plates were subsequently photographed and examined using a stereomicroscope. The captured images were imported into ImageJ software (https://imagej.net/ij/), and the region of interest was selected. Images were converted to 8-bit, and parameters including threshold and size range were adjusted to enable colony detection and area measurement. Colony size was expressed in mm^2^.

### Pigment extraction

After incubation under light and dark conditions, the cultures were collected to measure the optical density at 600 nm (OD600). Following homogenization, cells were harvested by centrifugation (4,500 × *g* for 20 min at 4°C), washed twice with 1 × PBS, and the resulting cell pellets were extracted with 100% methanol. Pigment extraction was performed by shaking the cell-methanol mixture overnight at 25°C and 180 rpm in centrifuge tubes wrapped with aluminum foil. To preliminarily identify the pigments, the methanol extract was subjected to full-wavelength scanning using a Cary 60 UV-Vis spectrophotometer (Agilent Technologies, Santa Clara, CA, USA). For pigment quantification, the absorbance of the methanol extract was measured at 437 nm (OD437) and then normalized to the total cell density (OD600 × culture vol) to calculate the specific absorbance intensity (ABS_437_/OD).

### Reactive oxygen species (ROS) quantification

Intracellular ROS levels were assayed using the cell-permeable probe 2′,7′-dichlorodihydrofluorescein diacetate (DCFH-DA; Sigma-Aldrich), following the established protocol (24). After incubation under light and dark conditions, the bacterial culture was transferred to a centrifuge tube with the DCFH-DA probe added to a final concentration of 150 μΜ. After mixing by gentle inversion, the mixture was incubated at 20°C for 30 min to allow the diffusion of the probe into bacterial cells. Cells were then collected by centrifugation (4,500 × *g* for 10 min at 4°C), washed, and suspended with 500 μL 1× PBS. We quantified intracellular ROS with an excitation wavelength of 488 nm and an emission wavelength of 526 nm to detect the fluorescent intensity. Relative fluorescence units (RFUs) were normalized to cell density (OD600 nm), and the results were expressed as specific fluorescence intensity (RFU/OD600).

## RESULTS

### Enrichment and isolation of deep-sea bacteria with light-promoted growth

During sampling, a white biofilm was observed on the surface of the chimney fragments, with numerous sea anemones and shrimp observed in the surrounding area (Fig. 1A). Samples were subsequently incubated in the laboratory under 30 MPa with illumination, using custom-designed HPI incubators (Fig. 1A). To improve isolation efficiency for light-responsive microorganisms, we selected a low-nutrient medium for anaerobic cultivation. After 1 month of incubation, the optical density in the illuminated reactor was higher than that in the dark control, indicating that light promoted microbial growth. Subsequently, the culture from the light-incubated reactor was spread onto solid plates. The plates were then randomly divided into two groups and grown at 0.1 MPa: one group was incubated under light, and the other was kept in the dark (Fig. 1A). As shown in Fig. S1, the plates exposed to light exhibit a greater number and larger size of colonies than those incubated in the dark. These colonies were further purified by streaking and identified by 16S rRNA gene sequencing. The growth curves were then determined under both light and dark conditions to identify strains with a light-promoted growth phenotype.

A total of 12 strains exhibiting light-promoted growth were obtained, belonging to four genera: *Microbacterium*, *Stutzerimonas*, *Achromobacter*,and *Pseudomonas* (Table S1). Among these, strains of *Microbacterium* and *Stutzerimonas* produced yellow pigments, whereas *Achromobacter* and *Pseudomonas* isolates did not (Fig. S2). The light-promoted growth phenotype was observed during the logarithmic growth phase in most strains, while no significant difference in biomass was detected during the stationary phase (Fig. S2). Notably, *Microbacterium* strains exhibited higher biomass and a more pronounced light-promoted growth phenotype, potentially as a result of pigment production. One such *Microbacterium* isolate, designated SY138, was selected for further investigation of the mechanism underlying light-promoted growth.

The 16S rRNA gene analysis indicated that strain SY138 belongs to the genus *Microbacterium* within the phylum *Actinobacteria*. Genome sequencing showed that strain SY138 has a circular chromosome of 3.92 Mb, a G+C content of 68.5%, and encodes 3,715 protein-coding genes. Average nucleotide identity (ANI) and digital DNA–DNA hybridization (dDDH) values between strain SY138 and its closely related type strains revealed the highest similarity to *Microbacterium liquefaciens* JCM 3879, with ANI and dDDH values of 88.81% and 35.5%, respectively (Table S2). Both values fall below thresholds for species delineation (ANI <95%–96%, dDDH <70%), indicating that strain SY138 represents a new species within the genus *Microbacterium*, which we propose to name *Microbacterium* sp. SY138.

### Light promoted the growth of strain SY138

To analyze the effect of light on the growth of strain SY138, we inoculated the strain into 2216E liquid medium and cultured it at 0.1 MPa under both light and dark conditions. As shown in Fig. 1B, the light-promoted growth phenotype was most pronounced during the exponential phase. At approximately 25 h, the biomass difference between the two conditions peaked, with an OD600 difference (light minus dark) of 0.29, representing a 1.69-fold increase relative to the dark control. Moreover, the growth rate of the light-cultured group was 0.03 h^−1^, while that of the dark-cultured group was 0.02 h^−1^, indicating that light exposure enhanced the growth rate of the SY138 strain. To further investigate the effect of light on growth at the colony level, we spread strain SY138 onto solid 2216E medium plates. The plates were randomly divided into two groups and incubated under light or dark conditions. As shown in Fig. 1C, the number of colonies did not differ significantly between light and dark plates, while colony size was visibly larger on the light-exposed plates. Quantitative analysis of colony areas revealed that the average colony size under light was 3.11 ± 0.89 mm^2^, compared to 1.68 ± 0.23 mm^2^ under dark conditions (Fig. 1D). These data indicate that colonies in the light group were significantly larger (*P* < 0.0001). Collectively, these results demonstrate that light enhances the growth of strain SY138 at both the population and colony levels.

### Light induced carotenoid production of strain SY138

**Fig 2:**
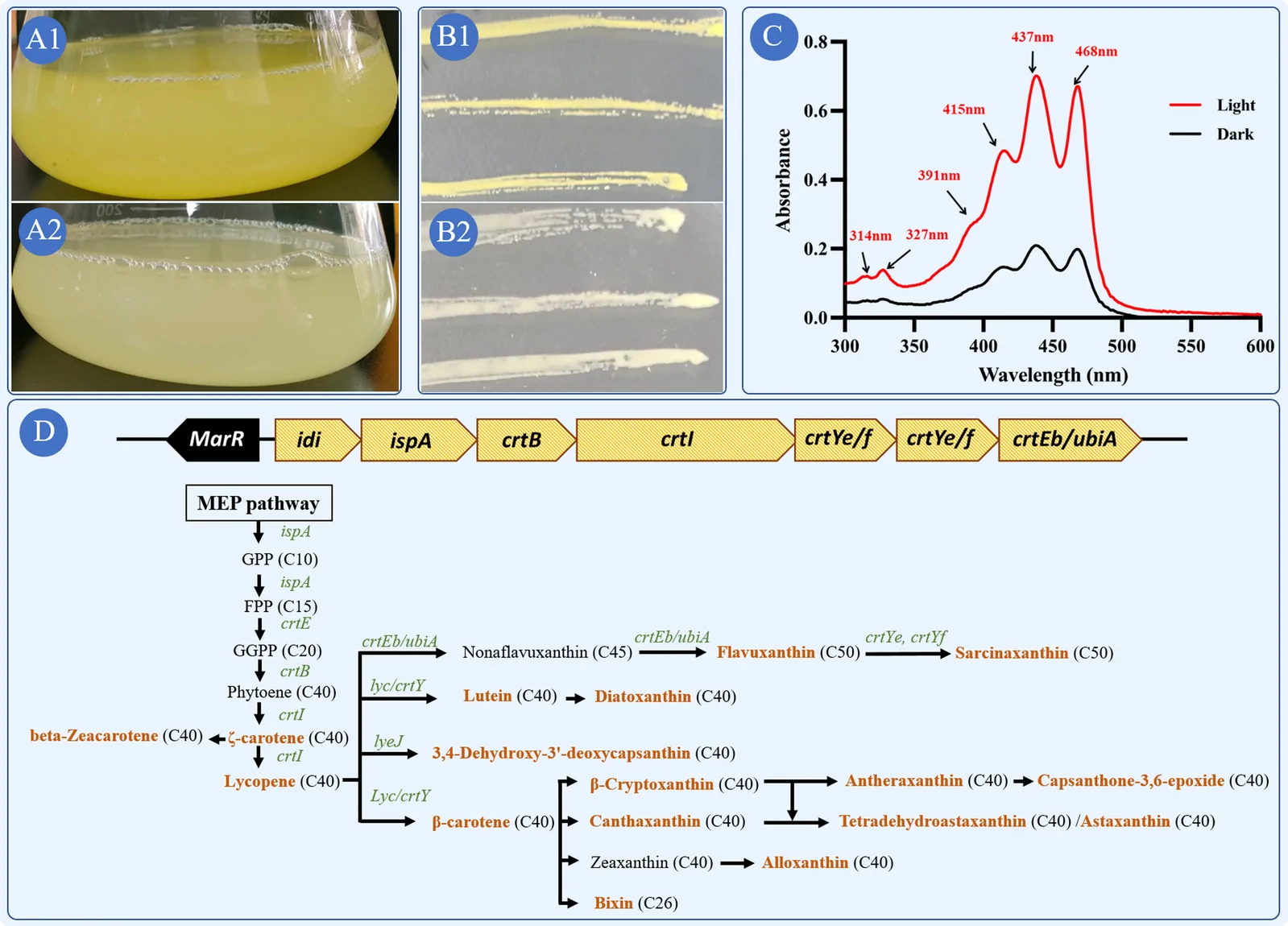
Light-induced pigment production and its biosynthetic pathways. (**A** and **B**) Light induced the production of yellow pigments by strain SY138 in both liquid (**A1** and **A2**) and solid media (**B1 **and **B2**). (**C**) UV-visible absorption spectra of pigments from strain SY138 under light and dark conditions. (**D**) The predicted carotenoid biosynthesis pathway in strain SY138. Green italic fonts represent enzyme-coding genes responsible for each catalytic step; orange-yellow fonts indicate carotenoids detected by LC-MS in this study; gray fonts refer to theoretical intermediates that were not detected via LC-MS; the number following C denotes the carbon atom number of the corresponding carotenoid skeleton.

To further investigate the mechanism underlying the light-enhanced growth, we analyzed the genome of strain SY138. However, no genes encoding bacteriochlorophyll, rhodopsin, or bacteriophytochrome were detected. This observation ruled out light-driven energy conversion as a primary mechanism for the observed growth promotion. During cultivation, colonies grown under light conditions developed a deeper color, whereas those cultured in the dark remained lighter (Fig. 1C1 and C2), suggesting that light induces pigment production in this strain. To test this possibility, we inoculated SY138 into the liquid medium (Fig. 2A1 and A2) and solid medium (Fig. 2B1 and B2) and incubated at 0.1 MPa under light or dark conditions. Light induced pigment production in both liquid and solid cultures. To further analyze the effect of light on pigment synthesis, we cultured SY138 under light or dark conditions and harvested cells at the stationary phase for pigment extraction and spectral analysis. As shown in Fig. 2C, the absorption profiles of pigments from both conditions were similar, with characteristic peaks at 314, 327, 391, 415, 437, and 468 nm, resembling the typical absorption spectrum of carotenoids. Quantification based on the main absorption peak at 437 nm (OD437) showed that the pigment yield per unit cell (OD437/OD600) was 0.13 under light and 0.04 in the dark, representing a 3.35-fold increase upon light exposure. These results confirm that light induces pigment production in strain SY138.

To delineate the genetic basis of light-induced pigment synthesis, we performed whole-genome analysis of strain SY138. The genome contains a complete methylerythritol phosphate (MEP) pathway for terpenoid backbone biosynthesis, which provides precursors for carotenoids. It also encodes key enzymes involved in carotenoid biosynthesis, including isopentenyl-diphosphate delta-isomerase (Idi), polyprenyl synthetase (IspA), phytoene synthase (CrtB), phytoene desaturase (CrtI), lycopene cyclase (CrtY), and prenyltransferase (CrtEb/UbiA). These genes are organized into a gene cluster, upstream of which lies an MarR-family transcriptional regulator (Fig. 2D). Furthermore, we used UPLC-Q-TOF/MS to identify the major carotenoid components of strain SY138. As shown in Table S3, a total of 18 carotenoids were detected. Among them, ζ-carotene was the most abundant, accounting for 58.6% of the total carotenoid content, followed by β-carotene at 22.3%. Based on these results, we reconstructed the carotenoid biosynthesis pathway for strain SY138 (Fig. 2D).

### Light reduces the growth inhibition caused by HHP

**Fig 3:**
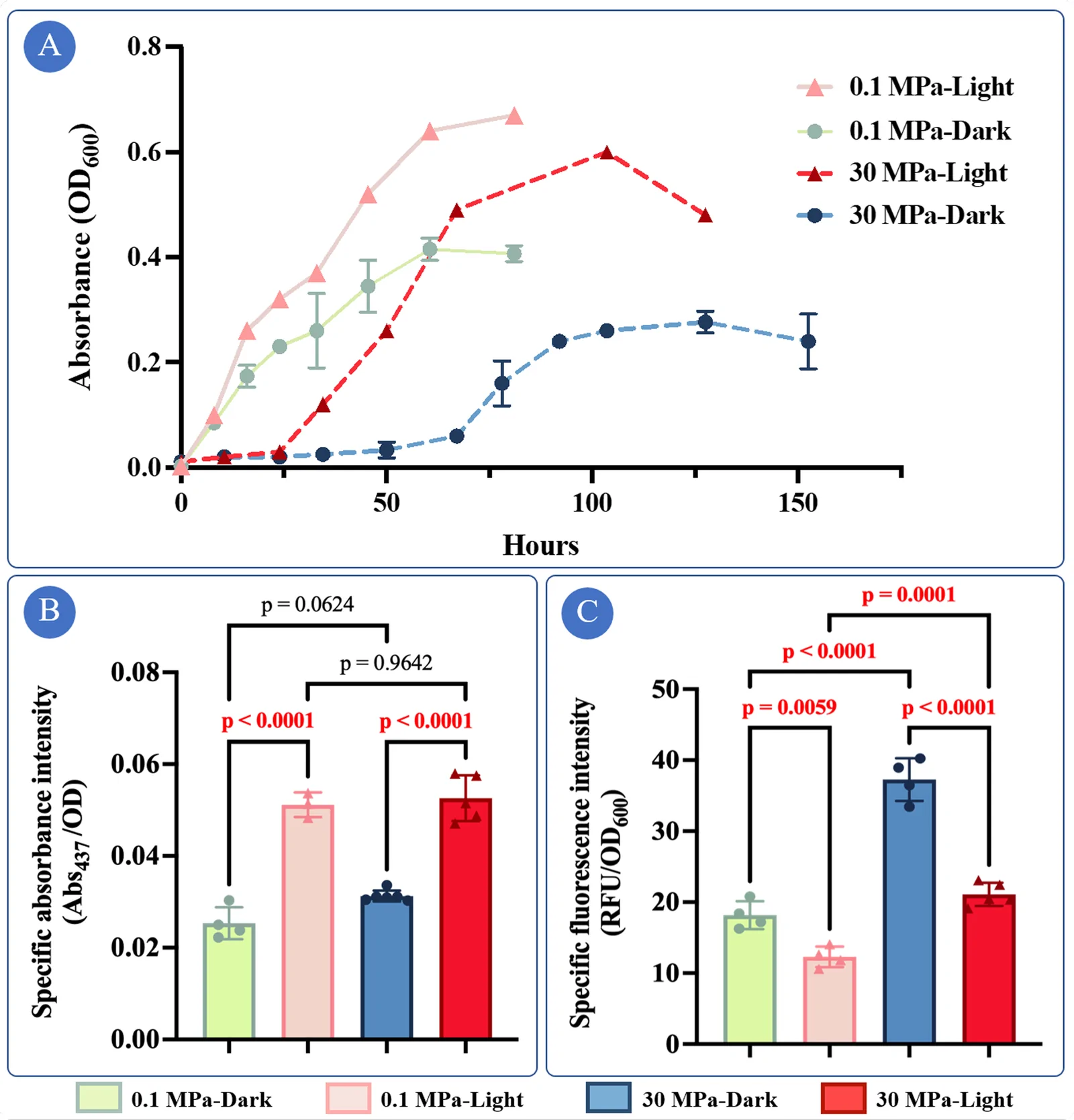
Effects of light exposure under HHP conditions on the *Microbacterium* sp. SY138. (**A**) Growth curves of strain SY138 under light and dark conditions at 0.1 MPa and 30 MPa, respectively. (**B**) Carotenoid production of strain SY138 under different conditions. (**C**) Intracellular ROS levels of strain SY138 under different conditions. For statistical analysis, one-way ANOVA with Šidák multiple comparisons test was used.

To investigate the effect of light on growth under HHP conditions, we constructed a high-pressure light-coupled cultivation system using a series of custom-designed high-pressure devices. As shown in Fig. 3A, at 0.1 MPa, the maximum biomass of the dark-cultured strain was OD600  =  0.42, while the light-cultured strain reached OD600  =  0.67, indicating that light significantly enhanced biomass accumulation. At 30 MPa, the maximum biomass of the dark-cultured strain was OD600  =  0.28, with a growth rate of 0.007. In contrast, the light-cultured strain achieved a maximum biomass of OD600 = 0.60, with a growth rate of 0.011. Under 30 MPa, light treatment resulted in an approximately 2.14-fold increase in maximum biomass compared to the dark culture. These results demonstrate that HHP inhibits growth, while light can effectively alleviate the growth-suppressing effect of HHP.

To further investigate the mechanism by which light alleviates HHP-induced growth inhibition, we measured carotenoid production under different culture conditions. As shown in Fig. 3B, at 0.1 MPa, the carotenoid yield was 0.025 ± 0.004 in the dark and increased to 0.051 ± 0.003 (Abs437/OD) under light. At 30 MPa, the corresponding yield was 0.031 ± 0.001 in the dark and 0.053 ± 0.005 under light. These data demonstrate that light significantly promotes carotenoid synthesis under both ambient and HHP conditions. We further assessed intracellular ROS levels under the same culture conditions (Fig. 3C). In the dark, the ROS level was 18.16  ±  1.96 RFU/OD600 at 0.1 MPa and increased to 37.27 ±  3.00  RFU/OD600 at 30 MPa, confirming that HHP markedly elevates ROS accumulation. In contrast, light exposure substantially reduced ROS levels to 12.29 ±  1.46 RFU/OD600 and 21.10 ±  1.64  RFU/OD600 at 0.1 MPa and 30 MPa, respectively. Collectively, these findings indicate that HHP inhibits growth primarily through increased ROS production, whereas light counteracts this effect by enhancing carotenoid synthesis and lowering intracellular ROS levels.

### The light-promoted growth of strain SY138 is dependent on carotenoids

**Fig 4:**
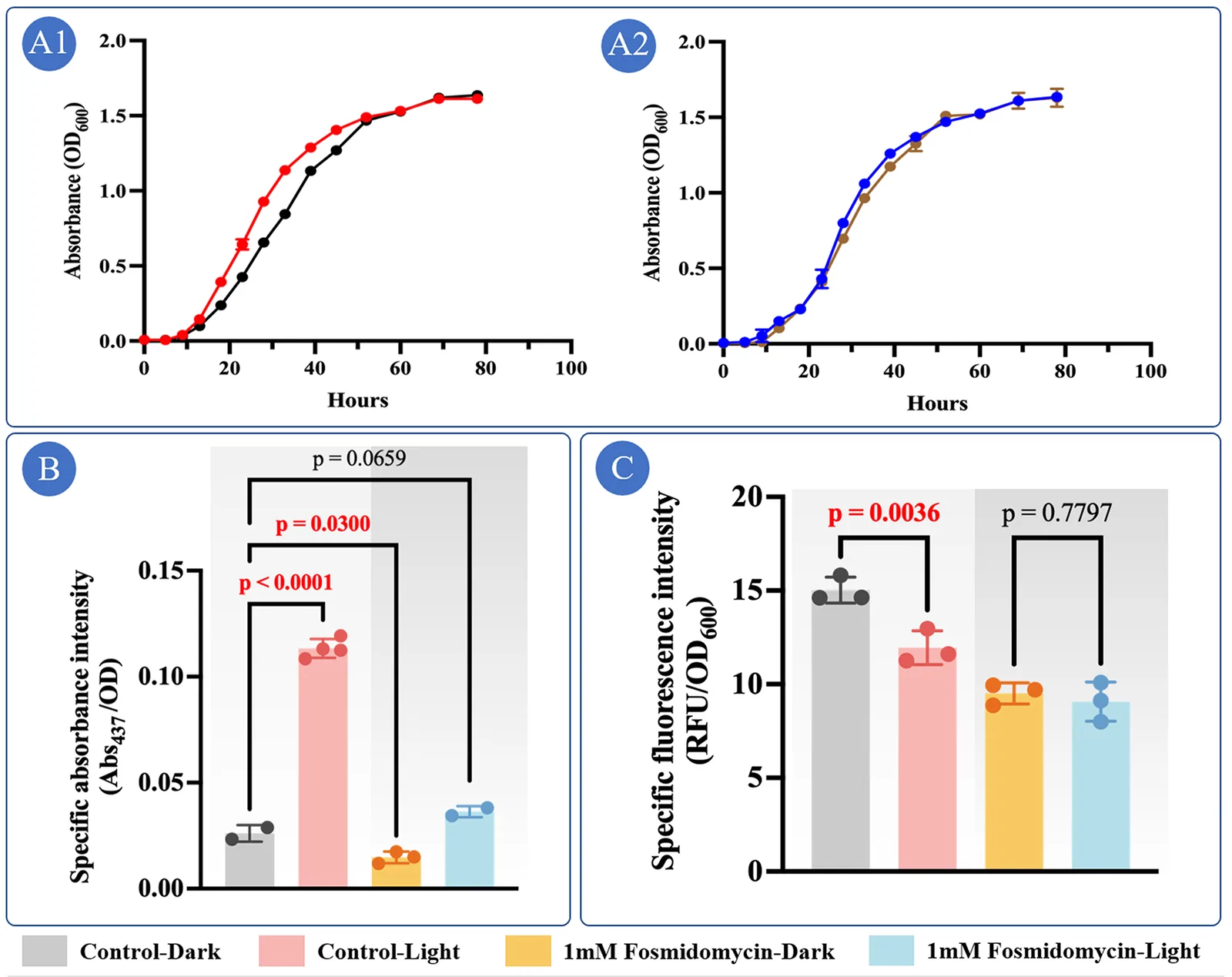
Effects of fosmidomycin on growth, carotenoid production, and intracellular ROS levels in strain SY138. (**A**) Growth curves of the strain SY138 at 0.1 MPa under light and dark conditions without (**A1**) and with (**A2**) fosmidomycin. (**B**) Carotenoid production of strain SY138 under different conditions. (**C**) Intracellular ROS levels of strain SY138 under different conditions. For statistical analysis, one-way analysis of variance (ANOVA) with Šidák multiple comparisons test was used.

Carotenoids are common antioxidant compounds (25). To investigate their role in strain SY138, we added fosmidomycin to the medium to inhibit carotenoid synthesis by targeting 1-deoxy-D-xylulose-5-phosphate reductoisomerase (Dxr), a key enzyme in the MEP pathway (26). We then compared bacterial growth, pigment yield, and intracellular ROS levels. As shown in Fig. 4A1, without the inhibitor, strain SY138 consistently exhibited a light-promoted growth phenotype during the exponential phase (13 h to 52 h), with the effect most pronounced at around 33  h, when the maximum biomass difference *Δ*OD600 (light-dark) reached 0.3. However, in the presence of 1 mM fosmidomycin, the light-promoted growth phenotype was completely abolished (Fig. 4A2).

Quantification of carotenoid production showed that in the absence of fosmidomycin, the carotenoid yield under dark conditions was 0.026 ± 0.004, while under light conditions, it was 0.113 ± 0.0045 in Abs437/OD units, representing a 4.35-fold increase under light. Upon addition of fosmidomycin, carotenoid yield dropped to 0.015 ± 0.0028 in the dark and to 0.036 ± 0.0026 in the light, indicating that fosmidomycin significantly inhibited carotenoid synthesis. Furthermore, we measured intracellular ROS levels under the same culture conditions. Without fosmidomycin, the intracellular ROS level in dark-cultured cells was 15.02 ± 0.69 RFU/OD600, compared to 11.95 ± 0.90 RFU/OD600 in light-cultured cells, demonstrating that light significantly reduced intracellular ROS. Upon fosmidomycin addition, no significant difference in intracellular ROS levels was observed between dark and light cultures (light: 9.07 ± 1.05 RFU/OD600; dark: 9.51 ± 0.56 RFU/OD600). In addition, we note that fosmidomycin treatment reduced the baseline ROS level in the dark from 15.02 to 9.51 RFU/OD600. However, the difference between light and dark was virtually eliminated (from 3.08 to 0.44), while carotenoid synthesis was simultaneously suppressed. This supports the interpretation that the loss of ROS reduction upon fosmidomycin addition is primarily due to the blockade of carotenoid biosynthesis. Collectively, these findings indicate that light promotes bacterial growth by inducing carotenoid synthesis, which in turn lowers intracellular ROS levels.

**Fig 5:**
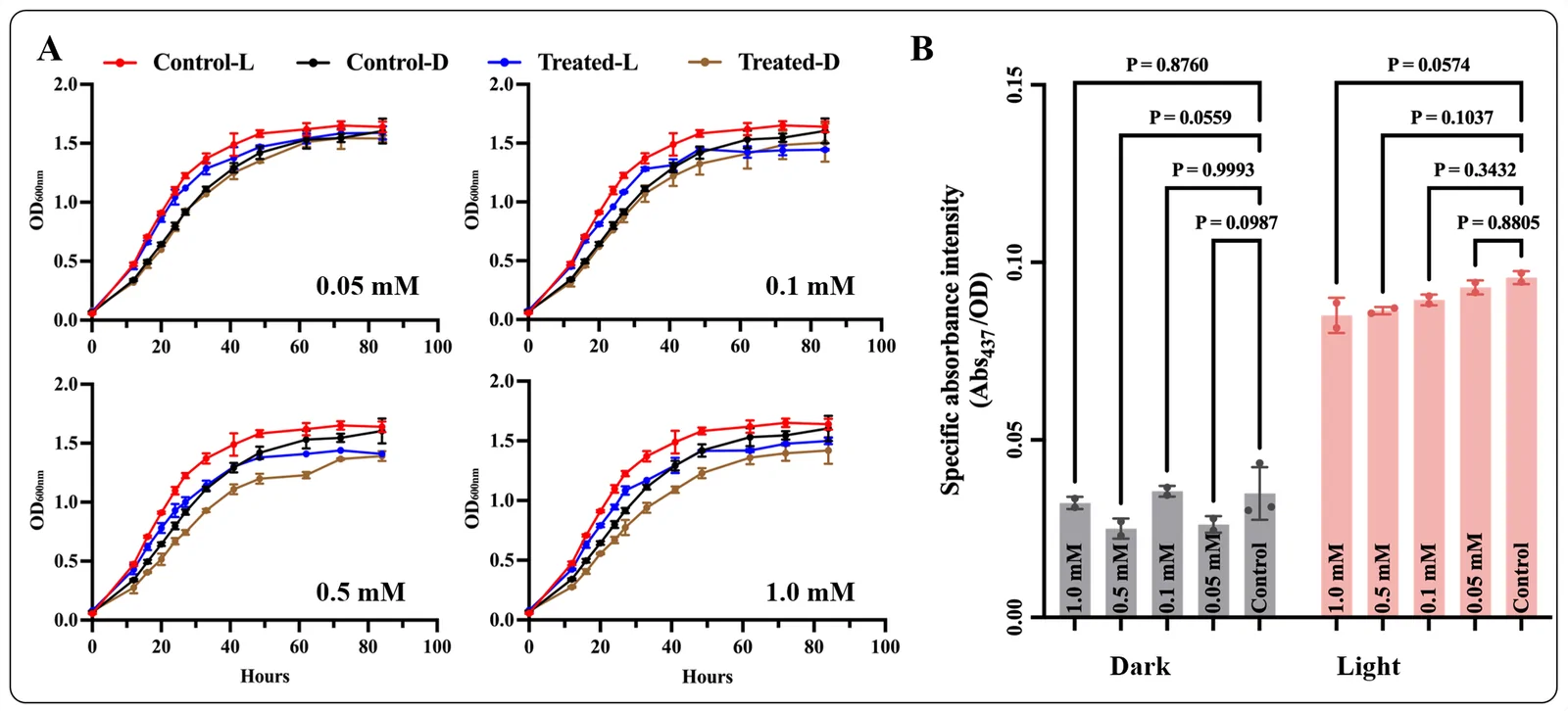
Effects of different concentrations of MPTA on the growth (**A**) and carotenoid production (**B**) of strain SY138. “L” refers to the light culture condition, and “D” refers to the dark culture condition. For statistical analysis, one-way ANOVA with Šidák multiple comparisons test was used.

In the crude carotenoid extract of the light-treated group, ζ-carotene accounted for more than half of the total carotenoid content. To further clarify the role of ζ-carotene, we added 2-(4-methylphenoxy)-triethylamine (MPTA) to the medium to inhibit lycopene cyclase (27), thereby interfering with the conversion of lycopene to downstream carotenoids. As shown in Fig. 5, the light-promoted growth phenotype did not strictly depend on the synthesis of downstream cyclic carotenoids as the phenotype persisted even when lycopene cyclization was blocked. Notably, MPTA treatment did not significantly alter total carotenoid content compared to untreated controls (Fig. 5B), suggesting that cells may maintain the pool of upstream precursors through feedback regulation. Collectively, these findings indicate that the antioxidant function is likely carried out by upstream acyclic precursors, with ζ-carotene being a prime candidate given its dominance in the pigment profile.

## DISCUSSION

Deep-sea environments, particularly hydrothermal vent ecosystems, are characterized by extreme conditions, including HHP, darkness, and chemical gradients. Although sunlight does not reach these environments, they are not entirely devoid of light; thermal radiation from geological processes provides a continuous source of photons (28, 29). The discovery of light-driven energy conversion mechanisms in deep-sea bacteria, such as bacteriochlorophyll-based photosynthesis and proteorhodopsin-based phototrophic metabolism, has challenged the conventional view that these ecosystems are exclusively driven by chemosynthesis (30, 31). Recent investigations of yeast isolates from deep-sea hydrothermal vents have demonstrated that infrared light stimulates growth through the upregulation of mitochondrial oxidative phosphorylation and ribosomal activity, thereby extending the recognized energetic framework of hydrothermal vent ecosystems beyond conventional chemosynthesis and photosynthesis (32). In this study, using a targeted enrichment strategy based on high-pressure incubation (30 MPa), we successfully isolated 12 bacterial strains with light-promoted growth phenotypes, among which *Microbacterium* sp. strain SY138 was selected for further mechanistic investigation due to its pronounced light-responsive phenotype. Notably, genomic analysis revealed no genes associated with canonical phototrophic systems (e.g., bacteriochlorophyll or rhodopsins), ruling out light-driven energy conversion as the basis for the observed growth promotion. Instead, the conspicuous yellow pigmentation induced by light pointed to a photoprotective or stress-alleviating mechanism.

HHP in deep-sea environments represents a major physical stressor known to elevate intracellular ROS levels (24, 33). In this study, HHP significantly increased intracellular ROS levels and inhibited the growth of strain SY138, consistent with previous reports showing HHP disrupts microbial metabolic homeostasis and induces oxidative stress in piezophilic and piezotolerant bacteria. Importantly, light reversed this effect; it reduced intracellular ROS levels and restored growth under HHP to levels comparable to those observed under atmospheric conditions (Fig. 3). Pigment analysis confirmed that light induced carotenoid production in strain SY138. Carotenoids are well known for their antioxidant properties (34, 35). The absorption spectrum of the extracted pigments exhibited characteristic peaks consistent with carotenoids (415, 437, and 468 nm), and whole-genome sequencing further identified a complete carotenoid biosynthesis gene cluster. To establish a direct causal relationship among carotenoid synthesis, ROS reduction, and the light-promoted growth phenotype, we used fosmidomycin, an inhibitor of the MEP pathway. Inhibition of carotenoid synthesis completely abrogated both the light-induced ROS reduction and the light-promoted growth phenotype (Fig. 4), providing direct evidence that the light-promoted growth depends on carotenoid production. Collectively, our findings uncover a novel survival strategy in which light serves not as an energy source but as an environmental signal that triggers the synthesis of protective carotenoids, thereby alleviating HHP-induced oxidative stress and promoting growth in this deep-sea *Microbacterium* isolate.

Among the light-induced carotenoids, ζ-carotene, an upstream intermediate of lycopene, was identified as the major component. We added MPTA to the medium to inhibit lycopene cyclase, thereby blocking the further conversion of lycopene to downstream carotenoids. Notably, MPTA treatment did not abolish the light-promoted growth phenotype nor did it significantly reduce total pigment content. Given that ζ-carotene accounted for more than half of the total carotenoid content in strain SY138 and has been reported to possess potent antioxidant activity and UV protection functions (36, 37), we propose that light-induced carotenoids, particularly the abundant ζ-carotene, function as inducible antioxidant defense mechanisms that help counteract the intrinsic oxidative stress caused by HHP in this strain.

To determine whether the light-induced carotenogenesis of strain SY138 is strain-specific or broadly adaptive among vent microbiota, we surveyed CrtB and CrtI homologs in 13 Indian Ocean vent metagenomes (10 from the Southwest Indian Ridge and three from the Central Indian Ridge) (Fig. S3). CrtI-like sequences appeared in 84.6% of samples and CrtB-like in 61.5%, suggesting that carotenogenesis genes are widespread. CrtI abundance was consistently higher than CrtB across positive samples, possibly reflecting differences in copy number, sequence coverage, or enzyme roles. However, amino acid identities to SY138 were low (22.5%–28.5%), so functional conservation remains uncertain; these homologs may perform distinct reactions or be regulated differently. Their broad distribution, nonetheless, implies that carotenoid biosynthesis is a general adaptive trait among vent bacteria, rather than a SY138-specific feature, and that this pathway may also be subject to light-responsive regulation. Heterologous expression and biochemical characterization will be essential to define their functions and their potential involvement in light-dependent stress protection.

### Conclusions

In summary, this study demonstrates that light, a factor conventionally disregarded in deep-sea environments, can act as a physiological cue for stress preconditioning in vent-associated bacteria. Light-induced carotenoid biosynthesis alleviates oxidative stress under high hydrostatic pressure, suggesting that photoperception confers a selective advantage on hydrothermal vent microorganisms. Although our results derive from a single isolate and controlled laboratory experiments, they raise new questions concerning the ecological prevalence of photosensory systems in deep-sea microorganisms and their potential contribution to fitness under chronic HHP stress. Further investigations are warranted to elucidate the genetic underpinnings of this response and to evaluate its distribution across various hydrothermal vent habitats.

## Data Availability

The complete genome sequence of strain SY138 has been deposited in GenBank under accession number CP155793 (BioSample SAMN41258225).

## SUPPLEMENTAL MATERIAL

Supplemental material aem.00658-26-s0001.docx Fig. S1 to S3; Tables S1 to S3.

ASM does not own the copyrights to Supplemental Material that may be linked to, or accessed through, an article. The authors have granted ASM a non-exclusive, world-wide license to publish the Supplemental Material files. Please contact the corresponding author directly for reuse.
