# Supplementary material for: Light-induced carotenogenesis enhances growth and high hydrostatic pressure tolerance in a deep-sea bacterium by reducing intracellular ROS
Source: Appl Environ Microbiol. 2026 Aug 28;92(9):e00658-26. doi: 10.1128/aem.00658-26 (PMC13599544; doi:10.1128/aem.00658-26)
Supplement: Supplementary file 1 [file aem.00658-26-s0001.docx]

**SUPPLEMENT MATERIAL**

**Supplementary Figure S1**

**Figure S1.** Colony morphology under light and dark conditions. To ensure that the light-treated and dark-treated groups had the same initial biomass and identical culture conditions, we took equal volumes of the light-treated group culture and spread them onto two separate plates. One plate was placed under light culture conditions, while the other was wrapped in aluminum foil to exclude light and placed under the same lighting conditions.

**Supplementary Figure S2**

**Figure S2.** Growth curves and colony morphologies of the 12 strains. "L" refers to the light culture condition, and "D" refers to the dark culture condition. The inset in each growth curve shows the colony morphology on plates cultured under light conditions, with the scale bar representing 5 mm.

**Figure S3.** CrtB and CrtI-like abundance dominates in several Central Indian Ridge and Old City samples. Sum Depth/Gbp by sample and query after total-bases normalization.

**Supplementary Table S1**

**Table S1.** Characteristics of strains with light-enhanced growth phenotypes

| ID | 16S rRNA gene sequence analysis | Identities (%) | Colony color |
| --- | --- | --- | --- |
| 1 | *Achromobacter anxifer* | 99.57 | White |
| 2 | *Achromobacter marplatensis* | 99.42 | White |
| 3 | *Microbacterium algeriense* | 99.86 | Yellow |
| 4 | *Microbacterium oxydans* | 100 | Yellow |
| 5 | *Microbacterium oxydans* | 100 | Yellow |
| 6 | *Microbacterium oxydans* | 99.93 | Yellow |
| 7 | *Microbacterium laevaniformans* | 99.56 | Yellow |
| 8 | *Microbacterium laevaniformans* | 99.56 | Yellow |
| 9 | *Pseudomonas aeruginosa* | 100 | White |
| 10 | *Stutzerimonas kunmingensis* | 99.43 | Orange |
| 11 | *Stutzerimonas kunmingensis* | 99.5 | Orange |
| 12 | *Stutzerimonas stutzeri* | 99.79 | Orange |

**Supplementary Table S2**

**Table S2.** Genome comparisons between strain SY138 and closely relatives

| Strains | ANI (%) | DDH (%) |
| --- | --- | --- |
| *Microbacterium liquefaciens* JCM 3879 | 88.81 | 35.5 |
| *Microbacterium maritypicum* DSM 12512 | 88.79 | 35.8 |
| *Microbacterium oxydans* DSM 20578 | 86.81 | 31.7 |

**Supplementary Table S3**

**Table S3.** Mass spectrometry identification results of carotenoids in strain SY138

| ID | Metabolite name | Formula | Reference m/z |
| --- | --- | --- | --- |
| 1 | Bixin | C_25_H_30_O_4_ | 395.22168 |
| 2 | beta-carotene | C_40_H_56_ | 537.45001 |
| 3 | beta-Zeacarotene | C_40_H_58_ | 538.45331 |
| 4 | zeta-Carotene | C_40_H_60_ | 541.47681 |
| 5 | 3,4-Dehydroxy-3'-deoxycapsanthin | C_40_H_54_O | 550.41998 |
| 6 | (2R)-beta,beta-Caroten-2-ol | C_40_H_56_O | 553.43402 |
| 7 | Alloxanthin | C_40_H_52_O_2_ | 565.40399 |
| 8 | Diatoxanthin | C_40_H_54_O_2_ | 566.40997 |
| 9 | (all-E)-Lutein | C_40_H_56_O_2_ | 569.00323 |
| 10 | 2,6-Cyclolycopene-1,5-diol | C_40_H_58_O_2_ | 571.45099 |
| 11 | Antheraxanthin | C_40_H_56_O_3_ | 584.41998 |
| 12 | Canthaxanthin (Euglenanone) | C_40_H_52_O_2_ | 587.38593 |
| 13 | 7,8,7',8'-Tetradehydroastaxanthin | C_40_H_48_O_4_ | 592.35999 |
| 14 | Astaxanthin | C_40_H_52_O_4_ | 596.39001 |
| 15 | Capsanthone-3,6-epoxide | C_40_H_54_O_4_ | 598.40002 |
| 16 | Flavuxanthin | C_50_H_72_O_2_ | 704.54999 |
| 17 | beta-Cryptoxanthin-palmitate | C_56_H_86_O_2_ | 790.99915 |
| 18 | Sarcinaxanthin monoglucoside | C_56_H_82_O_7_ | 866.60999 |
